# Evaluation of Dysphagia in Myositis and Muscular Dystrophy Using Real‐Time MRI and Quantitative Muscle Ultrasound

**DOI:** 10.1002/jcsm.70187

**Published:** 2026-03-13

**Authors:** Rachel Zeng, Anke Rietveld, Omar Al‐Bourini, Rosemarie H. M. J. M. Kroon, Arno Olthoff, Matthias Weidenmüller, Per‐Ole Carstens, Isabel Kommerell, Saskia G. Schütz, Corinne G. C. Horlings, Johanna G. Kalf, Bert J. M. de Swart, Baziel G. M. van Engelen, Tim Friede, Sabine Hofer, Jens Frahm, Ali Seif Amir Hosseini, Jens Schmidt, Christiaan G. J. Saris

**Affiliations:** ^1^ Department of Neurology, Neuromuscular Center University Medical Center Göttingen Göttingen Germany; ^2^ Department of Neurology Radboudumc Research Institute for Medical Innovation Nijmegen the Netherlands; ^3^ Department of Clinical and Interventional Radiology University Medical Center Göttingen Göttingen Germany; ^4^ Department of Rehabilitation Radboudumc Research Institute for Medical Innovation Nijmegen the Netherlands; ^5^ Department of Otorhinolaryngology, Phoniatrics and Pedaudiology University Medical Center Göttingen Göttingen Germany; ^6^ Department of Neurology Medical University Innsbruck Innsbruck Austria; ^7^ Department of Medical Statistics University Medical Center Göttingen Göttingen Germany; ^8^ Department of Neurology University Medical Center Göttingen Göttingen Germany; ^9^ Biomedical NMR Max Planck Institute for Multidisciplinary Sciences Göttingen Germany; ^10^ Else Kröner Fresenius Center for Optogenetic Therapies University Medical Center Göttingen Göttingen Germany; ^11^ Department of Neurology and Pain Treatment, Neuromuscular Center Immanuel University Hospital Rüdersdorf, Brandenburg Medical School Theodor Fontane Rüdersdorf Germany; ^12^ Faculty of Health Sciences Brandenburg Brandenburg Medical School Theodor Fontane Rüdersdorf Germany

**Keywords:** cricopharyngeal bar, imaging, neuromuscular disorders, quantitative MRI, swallowing muscles

## Abstract

**Background:**

Swallowing dysfunction—dysphagia—is a frequent and debilitating symptom in neuromuscular disorders, leading to malnutrition, cachexia, aspiration pneumonia, and death. Identification of the underlying pathophysiological mechanisms is important for diagnosis and treatment. As standard assessments have limitations, novel imaging techniques are needed. We here studied the utility of real‐time MRI and quantitative muscle ultrasound for characterizing dysphagia in two different neuromuscular disorders.

**Methods:**

This prospective cohort study included 18 patients with inclusion body myositis (IBM, 33% female, age 68.9 ± 7.7 years) and 13 with oculopharyngeal muscular dystrophy (OPMD, 62% female, age 55.9 ± 7.0 years) from two European Neuromuscular research centers (Nijmegen, NL; Göttingen, DE). Swallowing function was studied using real‐time MRI (RT‐MRI), FEES (flexible endoscopic evaluation of swallowing), and clinical assessments. T1‐mapping and quantitative muscle ultrasound (QMUS) were used to analyse tissue properties in swallowing muscles. Outcomes were compared between the two muscle diseases. RT‐MRI values were also compared with 22 age‐ and sex‐matched non‐myopathic controls.

**Results:**

RT‐MRI revealed significantly prolonged oral transit times in OPMD vs. controls (difference between means = 581.2 ms, 95% CI 225.9–936.4, *p* = 0.002). Pharyngeal transit time was significantly prolonged in IBM vs. controls (difference between means = 1132.8 ms, 95% CI 482.2–1783, *p* = 0.001). A cricopharyngeal bar as a well‐established morphological indicator of dysphagia was identified in 80% of patients with IBM compared with 53% in OPMD. Fatty degeneration of the tongue in OPMD significantly correlated between MRI‐T1 values and ultrasound echogenicity (Spearman's ρ = −0.52, *p* = 0.005). ROC revealed excellent discrimination between diseases by combining RT‐MRI, T1‐mapping and QMUS (AUC = 0.95, 95% CI 0.86–1.00), while FEES and clinical assessments failed to differentiate specific patterns of dysphagia.

**Conclusions:**

This study supports the value of novel MRI and ultrasound techniques for clinical use by identifying the pathophysiology and severity of impaired swallowing. Differentiating the phenotypes of dysphagia can aid in the diagnosis and treatment of affected patients. RT‐MRI and QMUS may serve as outcome measures for swallowing in clinical trials.

**Abbreviations**
BMI: body mass index
CI: confidence interval
CPB: cricopharyngeal bar
CT: computed tomography
EOT: oesophageal opening time
FEES: flexible endoscopic evaluation of swallowing
HMA: hyoid‐mental approximation
IBM: inclusion body myositis
LAT: laryngeal ascent
MRC: Medical Research Council
OPMD: oculopharyngeal muscular dystrophy
OTT: oral transit time
PCT: pharyngeal constriction time
PTT: pharyngeal transit time
QMUS: quantitative muscle ultrasound
ROC: receiver operating characteristic curves
ROI: region of interest
RT‐MRI: real‐time MRI (of swallowing)
SD: standard deviation
SWAL‐QoL: Swallowing‐Reated Quality of Life
UES: upper oesophageal sphincter
VF: videofluoroscopy

## 1 Introduction

Dysphagia can greatly affect quality of life and life expectancy in patients with neuromuscular disorders. Inclusion body myositis (IBM), an acquired inflammatory myopathy, and oculopharyngeal muscular dystrophy (OPMD), a hereditary myopathy, both typically affect patients at an advanced age, with dysphagia as one of the main symptoms, present in 65%–86% of IBM and 62%–100% of OPMD patients in the course of the disease [1, 2, 3].

The main mechanisms leading to dysphagia in IBM and OPMD are thought to be decreased pharyngeal pressure generation and impaired relaxation of the upper oesophageal sphincter (UES) [4, 5, 6], the latter often associated with the presence of a cricopharyngeal bar (CPB), a propulsion at the level of the UES, especially in IBM patients [1, 7, 8]. Controversies about the causes of dysphagia remain. Furthermore, treatment of UES dysfunction using cricopharyngeal dilatation, botulinum toxin injection, or myotomy frequently does not yield the desired effects and not all patients benefit from those treatments (reviewed in [9]). These observations suggest important differences in the mechanism of dysphagia in different diseases and even individual patients, which cannot be fully assessed using standard diagnostics such as flexible endoscopic evaluation of swallowing (FEES) and videofluoroscopy. As both IBM and OPMD are currently incurable muscle disorders with no established management of dysphagia in either disease, new diagnostic approaches are needed to improve understanding of the complex pathophysiology of dysphagia in order to optimize diagnosis and treatment.

Real‐time MRI (RT‐MRI) allows continuous visualization of physiologic swallowing at a high temporal resolution [10] and has been shown to be a reliable diagnostic tool for assessing dysphagia in IBM [2], but its utility for discriminating between different phenotypes of dysphagia remained an open question. In addition to dynamic imaging of swallowing, the quantification of morphological pathologies of the affected muscles is desirable. Ultrasound is increasingly used to detect fibrosis and fatty replacement of affected muscles including orofacial muscles, with quantitative muscle ultrasound (QMUS) offering the most sensitive approach by measuring muscle thickness and mean grayscale level and comparing them to normal values for the respective muscles, based on sex, age, length and weight, thereby minimizing potential bias by confounding effects from any of these parameters [11]. More recently, quantitative muscle MRI has been proposed for assessment of fatty infiltration in skeletal muscles [12]. In this study, we applied fast T1 mapping of orofacial muscles based on the RT‐MRI technique (T1FLASH) [13] to calculate T1 values, which decrease with the degree of fatty replacement of the muscle tissue [14].

These advanced imaging techniques have the potential to improve our understanding of complex conditions such as dysphagia. Therefore, the aim of this study is to evaluate the diagnostic value of novel real‐time MRI (RT‐MRI) and quantitative muscle ultrasound (QMUS) for the assessment of dysphagia in two different neuromuscular disorders.

## 2 Methods

### 2.1 Study Design and Participants

A total of 18 IBM and 13 OPMD patients were recruited from tertiary neuromuscular outpatient clinics: 2 IBM patients and 13 OPMD patients from Radboudumc, Nijmegen, The Netherlands and 16 IBM patients from University Medical Centre, Göttingen, Germany. IBM patients fulfilling the ENMC 2011 criteria of “clinicopathologically” or “clinically” defined IBM were included [15]. All OPMD patients had a confirmed PABPN1 mutation. Patients were recruited irrespective of presence or absence of dysphagia or other clinical parameters. None of the patients underwent cricopharyngeal muscle myotomy, botulinum toxin injection or dilatation prior to participation.

Exclusion criteria in both patient groups were (1) presence of diseases that might influence study results, including another neuromuscular or degenerative disorder that might impede swallowing, (2) significant intellectual impairment impeding the performance of testing, and (3) inability to lie in a supine position.

Two patients could not travel to the German study site; therefore, FEES and RT‐MRI were performed in 16 of 18 IBM patients.

### 2.2 Questionnaires

Each patient filled in the swallowing‐related quality of life questionnaire (SWAL‐QoL), consisting of 44 items. The score per item was calculated, resulting in a cumulated score ranging from 100% (no dysphagia‐related reduced quality of life) to 0% (major dysphagia‐related reduced quality of life) [S1, S2].

### 2.3 Clinical Assessments of Swallowing

Standard swallowing assessments were performed by an experienced speech therapist, including swallowing speed, volume test, bite force, tongue strength and tongue endurance, chewing time for a standardized cracker and phonation time (detailed descriptions are provided in Appendix S1). Results of the swallowing assessment were compared with normal values of age‐matched healthy controls from our local database [S3].

### 2.4 Flexible Endoscopic Evaluation of Swallowing (FEES)

FEES was performed by a masked specialist. Technical details are provided in Appendix S2. In addition to the Murray secretion scale (score 0–2) [S10] and Rosenbek's penetration‐aspiration scale (score 1 to 8) [S11], in which the lowest scores correspond to normal findings, the parameters leaking and retention (“yes” vs. “no”) were determined.

### 2.5 Quantitative Muscle Ultrasound

QMUS measurements were performed [11, 16] by a single researcher with the Esaote MyLabTwice ultrasound scanner (Esaote SpA, Genoa, Italy) using an 8–14 MHz broadband linear transducer with a 53‐mm footprint. Three consecutive ultrasound images were registered and analysed offline. A region of interest (ROI) was manually selected in each image with a custom software program for quantitative muscle image analysis developed at Radboudumc. The muscle thickness and the mean echogenicity of the ROI (i.e., histogram based grey‐scale analysis) were assessed for the geniohyoid muscle and tongue, and bilaterally for the anterior belly of the digastric muscle, masseter, and temporal muscle.

Previously published reference values of healthy subjects were used to calculate z‐scores (i.e., the number of standard deviations from the predicted normal value based on sex, age, length and weight); these reference values are not available for muscle thickness of the tongue, geniohyoid and digastric muscles. Echogenicity z‐scores between 1.5 and 2 were considered borderline abnormal and z‐scores ≥ 2 (i.e., above the 95th percentile) of at least one side (left or right) were considered abnormal; z‐scores > 3 (i.e., above the 99th percentile) were considered severely abnormal. Muscle thickness z‐scores of ≤ − 1.5 of at least one side were considered abnormal [11, 16].

### 2.6 Real‐Time Swallowing MRI

Dynamic MRI of swallowing in real‐time (RT‐MRI) was performed on a 3 T MRI system (Skyra, Siemens Healthcare, Erlangen, Germany) using the previously established real‐time MRI technique based on a highly undersampled radial fast low‐angle shot (FLASH) acquisition in combination with image reconstruction by regularized nonlinear inversion [10, 17, 18]. The total image acquisition time was 40 ms, which yielded a true temporal resolution of 25 fps. Sequence parameters were: repetition time 2.13 ms, echo time 1.31 ms, flip angle 8°, field of view 192 × 192 mm^2^. Successive T1‐weighted images were acquired with an in‐plane resolution of 1.5 × 1.5 mm^2^ and a slice thickness of 8 mm in a mid‐sagittal plane.

The examination was based on a previously established protocol [19, 20]. Patients were examined in a supine position. Pineapple juice with natural manganese as paramagnetic agent, thickened with starch (Quick& Dick, Pfrimmer Nutrica, Erlangen, Germany), was used as an oral contrast and filled into a 50 mL syringe (Original Perfusor Syringe, 50 mL, Braun, Melsungen, Germany). The syringe was connected to a catheter (Type Heidelberger, 150 cm, Braun, Melsungen, Germany) and the other end was placed in the mouth of the patient. Patients were given the opportunity to test swallowing pineapple juice in the supine position on the MRI table under direct medical supervision prior to the scan, and the scan was only performed if there was no clinical evidence of aspiration. After starting the dynamic image recording, a bolus of 10 mL was administered to the patient during the MRI scan and the patient was asked to swallow in a normal manner at a comfortable pace. Each study participant underwent at least three consecutive MRI scans, each involving a complete swallow of 10 mL of contrast agent. Depending on individual anatomy, additional scans were taken with slight adjustments of the scan coordinates in the sagittal plane (3–5 mm shifts) to cover the entire region of interest. The measurement that best covered the entire swallowing process and included the relevant anatomical landmarks was selected for subsequent analysis.

**FIGURE 1:**
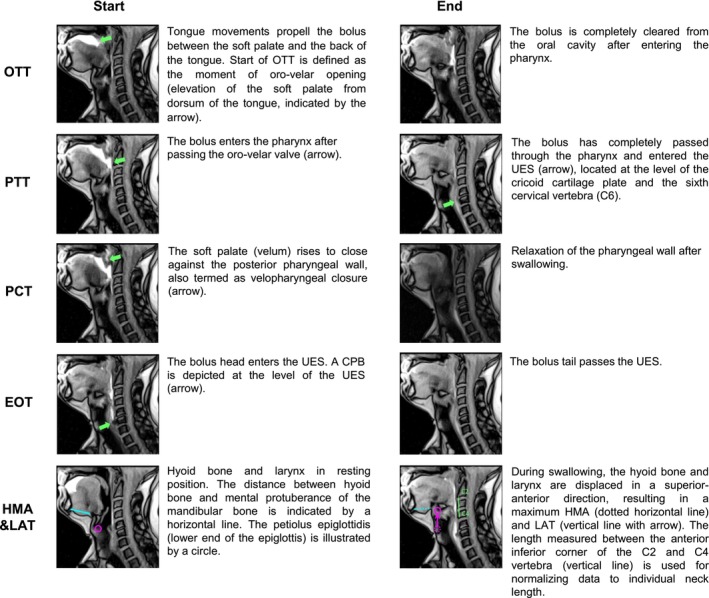
Frame‐by‐frame analysis of swallowing events in a 58‐year‐old male IBM patient as seen by real‐time MRI. Start and end points of distinct deglutition events on real‐time MRI are depicted as exemplary still images in the sagittal plane. Time measurements included the oral transit time (OTT), pharyngeal transit time (PTT), pharyngeal constriction time (PCT) and oesophageal opening time (EOT). Oral and pharyngeal transit times refer to the total time, measured in milliseconds, required for the subject to swallow the whole 10 ml bolus of administered contrast agent. The width of the upper oesophageal sphincter (UES), hyoid‐mental‐approximation (HMA) and laryngeal ascent (LAT) were measured in millimetres. If a cricopharyngeal bar (CPB) was observed, the width of the upper oesophageal sphincter was measured at the level of the CPB.

For quantitative evaluation of RT‐MRI, the OsiriX MD viewing software (http://osirix‐viewer.com) was used. Evaluations were performed by three specialists, who were masked for the patient's personal data and the results of other modalities. They independently evaluated the RT‐MRI data and subsequently reached consensus by joint reading. The evaluation was based on a modified version of a previously established protocol [2, 19, 20] (Figure 1). Start and end points of distinct deglutition events were analysed frame by frame in the sagittal view. Time measurements included the oral transit time, pharyngeal transit time, pharyngeal constriction time, and oesophageal opening time. Oral and pharyngeal transit times refer to the total time, measured in milliseconds, required for the subject to swallow the whole 10 mL bolus of administered contrast agent, which may include multiple swallowing attempts. Because the vallecula and piriformis sinus are not in the field of view on the mid‐sagittal imaging plane, residues in these areas were not assessed. The width of the upper oesophageal sphincter, hyoid‐mental approximation, and laryngeal elevation were measured in millimetres. If a cricopharyngeal bar (CPB) was observed, the width of the upper oesophageal sphincter was measured at the level of the CPB.

### 2.7 T1 Mapping

Measurement of T1 relaxation times enables quantitative assessment of tissue properties and their physiological and pathological alterations. The T1FLASH technique used here is based on a single‐shot inversion‐recovery method with serial acquisitions of highly undersampled radial FLASH images [13]. Sequence parameters were: repetition time 4.58 ms, echo time 2.86 milliseconds, flip angle 6°, field of view 192 × 192 mm^2^, resolution 0.5 × 0.5 mm^2^, slice thickness 4 mm, time per frame 69 ms.

To analyse muscle T1 values, regions‐of‐interest (ROI) were manually outlined around the individual muscles using OsiriX MD, which showed mean T1 values. T1 maps were obtained from the lower head region in sagittal and coronal planes. For analysis of the tongue muscle, the coronal sequences were chosen to avoid mapping of the lingual septum, which consists mainly of fibrous tissue. The masseter muscle was also analysed in the coronal plane, whereas the geniohyoid muscle was studied in the sagittal plane due to better visual separability from the tongue muscle.

The results of RT‐MRI and T1 mapping were compared with normal reference values of a control group consisting of 22 healthy subjects and non‐myopathic patients, who were treated at the University Medical Centre Göttingen for conditions not affecting swallowing function. Mean age of the control group was 52 ± 20 years with 40.9% females.

### 2.8 Statistical Analysis

Comparison between 2 groups was made by independent *t*‐test for continuous data and a Fisher's exact test for dichotomous data. ROC were used to analyse the effectiveness of different diagnostic methods in discriminating between 2 groups. Results of continuous variables are shown as mean ± standard deviation (SD) and range (minimum—maximum). Results of comparisons between groups are expressed as mean difference between groups ±95% confidence interval (CI). *p* values are adjusted for multiplicity using the Holm–Šidák method, significance is defined as *p* ≤ 0.05. Spearman's rank‐order correlation was used to assess associations between variables for monotonicity; hypotheses for the correlation coefficients being equal to 0 were tested and p‐values provided. Significance is defined as *p* ≤ 0.05. Statistical analyses were performed using the software GraphPad Prism 9 (San Diego, CA).

A sample size of 20 patients per group (IBM and OPMD) yields a power of 80% for the two‐sample *t*‐test at the usual two‐sided significance level of 5% if the standardized mean difference (Cohen's d) is 1.3. The sample size calculation was performed using nQuery 9 (version 9.2.1.0).

## 3 Results

### 3.1 Characterization of the Cohort

18 IBM and 13 OPMD patients were included. Patients' demographics are summarized in Table 1. IBM patients were significantly older compared with OPMD patients (68.9 ± 7.7 versus 55.9 ± 7.0 years, *p* < 0.001). Scores of the SWAL‐QoL were comparable in both cohorts (IBM 73.4 ± 13.5%, OPMD 77.0 ± 10.5%, *p* = 0.42).

**TABLE 1:** Patient baseline characteristics.

	IBM (*N* = 18)	OPMD (*N* = 13)	*p*
Gender: female	6 (33%)	8 (62%)	0.16
Age in years (mean (SD) [range])	68.9 (7.7) [57–82]	55.9 (7.0) [44–65]	< 0.001
Duration of symptoms in years (mean (SD) [range])	8.6 (6.1) [2–23]	8.4 (4.7) [1–17]	0.93
BMI (mean (SD) [range])	26.7 (4.9) [19–36]	27.0 (4.5) [21–35]	0.87
SWAL‐QoL (mean (SD) [range])	73.4% (13.5%) [53–93]	77.0% (10.5%) [61–93]	0.42

*Note:* Comparison between the two groups was made by independent *t*‐test.

Abbreviations: BMI, body mass index; SWAL‐QoL, swallowing‐related quality of life questionnaire.

### 3.2 Clinical Assessments and FEES

Pooled clinical assessments showed abnormal results of swallowing, chewing, and speaking in IBM and OPMD compared with healthy controls (Table S1), except for tongue endurance and bite force, which did not differ significantly from normal values. Comparison of clinical swallowing assessments yielded no significant differences between IBM and OPMD patients.

Assessment by FEES showed pathological findings in the majority of patients from both disease groups: secretion (IBM 66.6%, OPMD 84.6%), bolus retention in the pharyngeal tract (IBM 80%, OPMD 100%), and laryngeal penetration (mean Rosenbek scale 4.0 in both IBM and OPMD), yet no significant differences between the two groups were observed (Table S2). None of the patients showed signs of aspiration during FEES.

### 3.3 Quantitative Muscle Ultrasound

**FIGURE 2:**
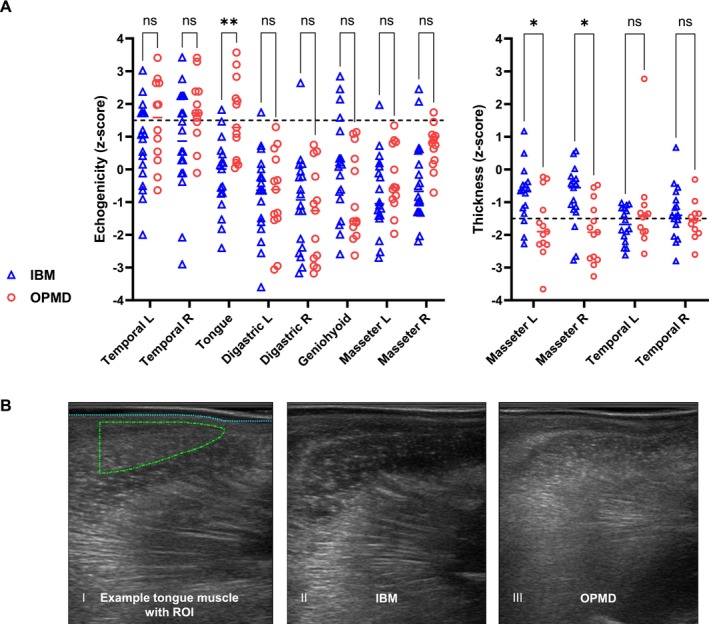
Quantitative muscle ultrasound (QMUS) of orofacial muscles in IBM and OPMD patients. A. Echogenicity and muscle thickness obtained by QMUS. IBM patients showed abnormal echogenicity, indicating fat replacement in various orofacial muscles except the tongue. In OPMD patients, the temporal muscle and tongue displayed severely abnormal echogenicity. Reduced thickness of the masseter and temporal muscles was found in both IBM and OPMD patients. Echogenicity z‐scores between 1.5 and 2 are considered borderline abnormal, z‐scores ≥ 2 are considered abnormal and z‐scores > 3 are considered as severely abnormal. Muscle thickness z‐scores ≤ − 1.5 are considered abnormal. Comparison between the two groups was made by independent t‐test, p‐values are adjusted for multiplicity using the Holm–Šidák method, significance is defined as *p* ≤ 0.05. B. Exemplary QMUS findings of the tongue muscle. Echogenicity was analysed using histogram based grey‐scale analysis and manually selected ROI, demarcated by the dotted green line (I), the dotted blue line delineates the border between the subcutaneous fat layer and muscle. Normal echogenicity of the tongue muscle in an IBM patient (II) and moderate signs of fibrosis and fatty infiltration in the tongue muscle of an OPMD patient (III).

The assessment of echogenicity and muscle thickness obtained by QMUS are shown in Figure 2. In IBM, the following orofacial muscles presented abnormal echogenicity (z‐scores of ≥ 2 of at least one side): temporal muscle (5/18 patients, 28%), geniohyoid muscle (3/18, 17%), masseter muscle (2/18, 11%) and digastric muscle (1/18, 6%). In OPMD, temporal muscle (6/12 patients, 50%) and the tongue (5/13, 38%) showed abnormal echogenicity. Severe abnormalities (z‐score > 3 of at least one side) were seen in temporal muscles in 2 IBM and 2 OPMD patients, and in the tongue of 2 OPMD patients. Reduced muscle thickness (mean z‐score ≤ − 1.5 of at least one side) of the masseter muscle was observed in 4/17 (24%) IBM patients and in 10/13 (77%) OPMD patients. Reduced muscle thickness of the temporal muscle was present in 10/17 (59%) IBM patients and in 7/12 (58%) OPMD patients. A heatmap showing individual results from QMUS is provided in Figure S1.

### 3.4 Real‐Time Swallowing MRI

**FIGURE 3:**
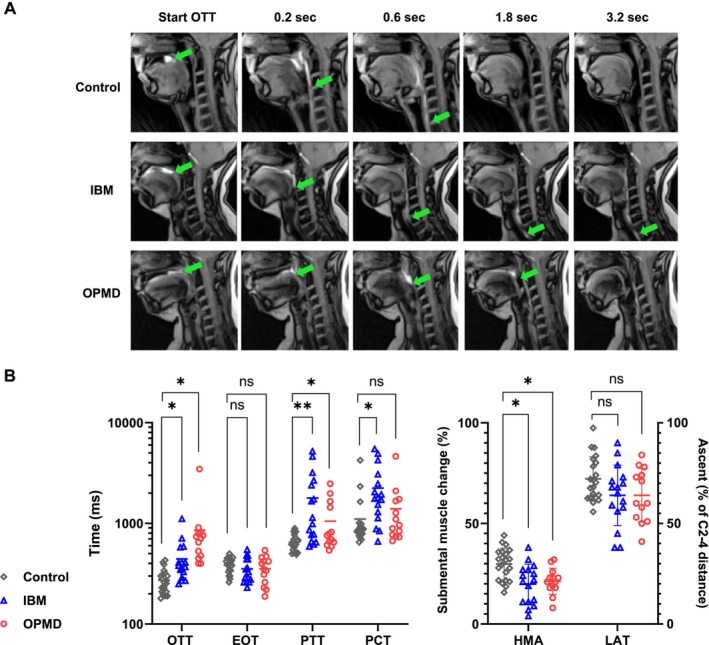
Real‐time MRI assessment of swallowing in patients with IBM and OPMD versus control subjects. (A) RT‐MRI at a resolution of 40 ms with exemplary findings in a control subject (upper row), an IBM patient (middle row) and an OPMD patient (bottom row). Images were selected at start of oral transit time (OTT) and subsequently 0.2, 0.6, 1.8, and 3.2 s. Note how swallowing one bolus of pineapple juice (bright contrast, bolus head is marked by a green arrow) is completed in the control subject at 1.8 s, while the IBM patient shows pharyngal retention and a cricopharyngeal bar at 1.8 and 3.2 s. The OPMD patient shows a prolonged oral phase of swallowing until 1.8 s after start of OTT, while the pharyngeal phase is unaffected and swallowing is completed by 3.2 s. These RT‐MRI sequences can be viewed in movie format in Movies S1 and S2. (B) RT‐MRI shows a significantly prolonged OTT in patients with OPMD and to a lesser extent also in patients with IBM. The pharyngeal phases of swallowing (PTT and PCT) are significantly impaired in patients with IBM and in a few patients with OPMD. No differences between the cohorts were found for oesophageal opening time (EOT). Hyoid‐mental approximation (HMA) is significantly reduced in both diseases. No differences to controls were observed for relative laryngeal elevation (LAT). Variables were tested using *t*‐test and compared with a non‐myopathic control group. *p* values were adjusted for multiplicity using the Holm–Šidák method, significance is defined as *p* ≤ 0.05.

Quantitative analysis of bolus transport by RT‐MRI (Table 2, Figure 3, Movies S1 and S2) showed prolonged oral transit times (OTT), significantly in OPMD (854 ± 801 ms) and to a lesser extent in IBM (440 ± 221 ms) when compared with the control group, where normal range of OTT was determined as 273 ± 74 ms. Pharyngeal transit time (PTT) and pharyngeal constriction time (PCT) were significantly prolonged in patients with IBM (PTT 1785 ± 1468 ms versus 653 ± 125 ms in controls; PCT 2243 ± 1471 ms versus 1102 ± 808 ms in controls). Some of the OPMD patients also displayed significantly prolonged PTT (1051 ± 628 ms), while the mean PCT showed no significant difference compared with controls. Oesophageal opening time (EOT) in both disease groups did not differ compared with controls.

**TABLE 2:** Quantitative analysis of real‐time swallowing MRI.

	IBM	OPMD	Controls	IBM vs. controls Mean difference (95% CI)	IBM vs. controls	OPMD vs. controls Mean difference (95% CI)	OPMD vs. controls
	Mean (SD)	Mean (SD)	Mean (SD)		*p*		*p*
OTT (ms)	440.4 (221.2)	853.7 (801.3)	272.5 (74)	167.9 (63.3–272.4)	**0.01**	581.2 (225.9–936.4)	**0.02**
PTT (ms)	1785.3 (1467.8)	1050.8 (628.1)	652.5 (124.8)	1132.8 (482.2–1783)	**0.009**	398.2 (112.8–683.6)	**0.05**
EOT (ms)	353.3 (100.5)	354.5 (108.8)	385.8 (67.8)	−32.5 (−90.4 to 25.3)	0.45	−31.39 (−93.9 to 31.1)	0.68
PCT (ms)	2242.5 (1470.7)	1393.8 (1074.9)	1102.4 (808.0)	1140.1 (372.0 to 1908)	**0.02**	291.5 (−367.9 to 950.8)	0.68
LAT abs. (mm)	23.6 (6.2)	22.9 (5.0)	26.5 (4.7)	−2.9 (−6.5 to 0.8)	0.31	−3.5 (−7.0 to −0.04)	0.22
LAT in relation to C2‐C4 distance (%)	64 (15.0)	64 (13.3)	72.1 (11.0)	−8.1 (−16.8 to 0.5)	0.24	−8.1 (−16.7 to 0.4)	0.23
HMA (%)	19.9 (9.9)	20.9 (6.0)	29.8 (7.9)	−10.0 (−15.9 to −4.0)	**0.01**	−8.6 (−13.9 to −3.3)	**0.02**
Oesophageal sphincter opening diameter (mm)	4.2 (0.7)	4.4 (0.8)	4.5 (0.9)	−0.3 (−0.9 to 0.3)	0.45	−0.13 (−0.8 to 0.5)	0.68

	IBM	OPMD			IBM vs. OPMD		
	Mean (SD)	Mean (SD)			*p*		
CPB occurrence	No = 3 Yes = 12	No = 6 Yes = 7			0.27		
CPB diameter (mm)	4.7 (1.8)	4.9 (1.5)			0.77		

*Note:* Quantitative analysis of bolus transport, laryngeal elevation, hyoid‐mental approximation, as well as occurrence and diameter of a cricopharyngeal bar. Variables were tested using *t*‐test, *p* values were adjusted for multiplicity using the Holm–Šidák method. Significance is defined as *p* ≤ 0.05. Data for oesophageal sphincter opening diameter and CPB not assessable in 1 IBM patient due to image artefacts. Bold values indicate statistical significance (p < 0.05).

Abbreviations: CPB, cricopharyngeal bar; EOT, esophageal opening time; HMA, hyoid‐mental approximation; LAT, laryngeal elevation; OTT, oral transit time; PCT, pharyngeal constriction time; PTT, pharyngeal transit time.

As a parameter for submental muscle constriction, maximum hyoid‐mental approximation (HMA) during swallowing was significantly reduced in IBM and OPMD compared with controls. Laryngeal elevation (LAT) was determined in absolute values as well as in relation to the distance between the second and fourth cervical vertebra (C2–C4); while the absolute LAT in OPMD patients was marginally reduced compared with controls, the values of the relative LAT did not reach statistical significance when comparing IBM and OPMD to healthy controls.

A cricopharyngeal bar (CPB) was identified via RT‐MRI in 12/15 IBM patients and in 7/13 OPMD patients. Due to imaging artefacts, CPB and the UES could not be analysed in one IBM patient. There was no significant difference in CPB diameter or UES opening diameter between the two disease groups. Although there was no significant statistical correlation between the diameter of the CPB with the PTT, all IBM patients with prolonged PTT displayed a CPB, while the only three IBM patients without CPB had a PTT in range of the controls (Spearman's correlation PTT and CPB occurrence *r* = 0.54, *p* = 0.05). For OPMD patients, neither the diameter nor the occurrence of a CPB correlated with the PTT.

### 3.5 T1 Mapping

**FIGURE 4:**
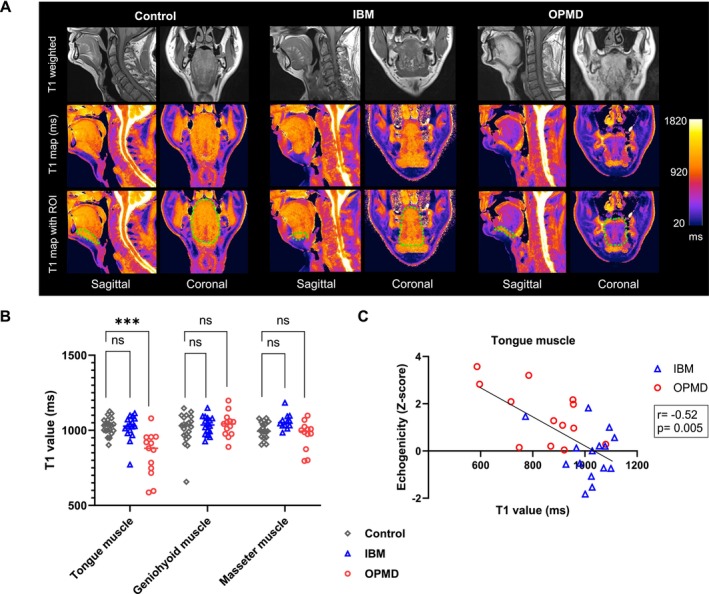
T1 mapping of swallowing muscles in patients with IBM and OPMD versus control subject. (A) Selected T1 weighted MRI sequences (upper row) and T1 maps (middle and bottom row) obtained for head and neck region of a control subject, an IBM patient and an OPMD patient, all male and between 61 and 71 years old. The bottom row outlines the manually placed regions of interest (dotted green lines) of the tongue muscles in the coronal sequences and the geniohyoid muscles in the sagittal sequences for analysis of mean T1 values. (B) T1 mapping reveal significantly lower T1 values of tongue muscle in OPMD patients, indicating a higher fat content. T1 values of geniohyoid muscle and masseter muscle show no significant differences between patients and controls. Variables were tested using *t*‐test. *p* values were adjusted for multiplicity using the Holm–Šidák method, significance is defined as *p* ≤ 0.05. (C) The reduced T1 values of the tongue in quantitative MRI correlate significantly with increased echogenicity of the tongue on ultrasound in OPMD patients (Spearman's *r* = −0.52; *p* = 0.005), both methods thereby indicate fibrosis and fatty infiltration of the tongue muscle.

T1 mapping showed significantly decreased mean T1 values of the tongue muscle, which indicates a higher fat content, in OPMD patients (843 ± 148 ms) compared with IBM patients (1018 ± 83 ms) (OPMD vs. IBM: difference between means = −175.0, 95% CI −264.1 to −85.87, *p* = 0.001) and controls (1027 ± 57 ms) (OPMD vs. controls: difference between means = −183.8, 95% CI −256.4 to −111.2, *p* ≤ 0.001) (Figure 4A,B). A significant correlation between decreased T1 values and increased echogenicity obtained by QMUS for tongue muscles was observed (analysis including both disease groups: Spearman's correlation *r* = −0.52, *p* = 0.005) (Figure 4C).

The mean T1 values in geniohyoid muscles of the disease groups (IBM 1033 ± 61 ms; OPMD 1037 ± 81 ms) showed no statistical differences to controls (1009 ± 106 ms). Although the mean T1 values of masseter muscles did not differ significantly between disease groups (IBM 1052 ± 54 ms; OPMD 980 ± 81 ms) and controls (999 ± 55 ms), two individual OPMD patients displayed marked decrease of T1 values in their masseter muscle down to 796 ms and 875 ms respectively. These two patients also showed significantly reduced muscle thickness of masseter muscles on QMUS, but only one of them displayed borderline abnormality of muscle echogenicity.

### 3.6 Correlations

Correlations of clinical swallowing assessments with outcomes from MRI and QMUS showed significant correlation of prolonged OTT as assessed by RT‐MRI with reduced swallowing speed in patients with IBM (*r* = −0.54, *p* = 0.03). The prolonged PTT in IBM patients showed correlation trends towards reduced swallowing speed (*r* = −0.36, *p* = 0.17) and longer chewing time needed to eat a cracker (*r* = 0.49, *p* = 0.05). Interestingly, a longer chewing time was significantly correlated with the occurrence of a CPB in IBM patients (*r* = 0.63, *p* = 0.01).

In patients with OPMD, reduced anterior and posterior tongue strength was correlated with abnormal echogenicity (*r* = −0.66, *p* = 0.02 and *r* = −0.65, *p* = 0.02, respectively), as well as reduced T1 values of tongue muscles (*r* = 0.59, *p* = 0.04 and *r* = 0.56, *p* = 0.05, respectively).

### 3.7 Classification Analysis

**FIGURE 5:**
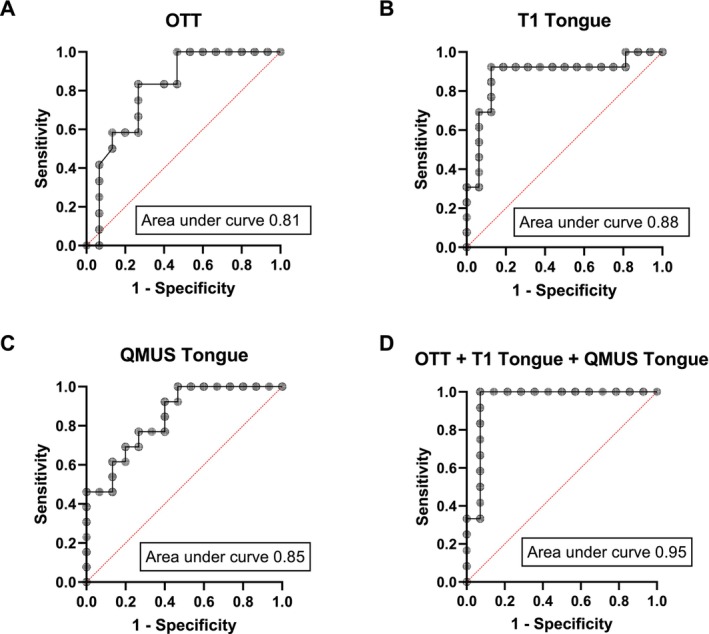
ROC curves for assessing the effectiveness of the different imaging techniques to discriminate between the two muscle diseases. ROC‐curves of sensitivity and specificity for differentiating between IBM and OPMD based on (A) OTT as seen by RT‐MRI, (B) T1 mapping of the tongue, (C) echogenicity of the tongue muscle as determined by QMUS, (D) combination of all three assessments.

The effectiveness of RT‐MRI, T1 mapping and QMUS in discriminating between dysphagia in IBM and OPMD were analysed using receiver operating characteristic curves (ROC) as shown in Figure 5. Maximum area under ROC of OTT as seen by RT‐MRI (AUC = 0.81; 95% CI 0.64–0.98), T1 value of tongue muscles (AUC = 0.88; 95% CI 0.74–1.00) and echogenicity of tongue muscles using QMUS (AUC = 0.85; 95% CI 0.71–0.99) were relatively high, with T1 values having the highest efficiency as a classifier. The highest classifying capabilities for discriminating between IBM and OPMD were observed for a combination of all three assessments (AUC = 0.95; 95% CI 0.86–1.00).

### 3.8 Complications

No serious adverse events were observed in this study.

## 4 Discussion

In this cohort study, we demonstrate the utility of novel MRI and quantitative ultrasound techniques in revealing distinct patterns of oropharyngeal involvement in different neuromuscular disorders. IBM patients primarily exhibit decreased pharyngeal motility and dysfunction of the UES on RT‐MRI, which is in line with previous reports [2, 7, 8]. Interestingly, this study also revealed signs of orofacial involvement in IBM in clinical assessments, QMUS and RT‐MRI. In OPMD, the most prominent findings were fatty degeneration of tongue muscles and an impaired oral transit time. Prolonged OTT included slow bolus transit as well as bolus fragmentation, the latter visible on RT‐MRI as multiple swallows of fragments of the given bolus during the oral phase of deglutition (Movies [Link], [Link]), most likely reflecting weakness of the muscles responsible for oral bolus control and formation, as well as an involuntary or trained behaviour in affected patients to avoid aspiration. In contrast, healthy subjects and patients without dysphagia usually swallowed the entire bolus of 10 mL contrast agent at once without fragmentation (Movie S3). Additionally, signs of decreased pharyngeal motility were shown by RT‐MRI in some OPMD patients. In both IBM and OPMD, PTT and PCT varied between individuals, with some IBM patients exhibiting exceptionally long pharyngeal transit times. This can be explained by differences in the severity of pharyngeal muscle weakness and, in the case of IBM, whether a prominent CPB is present. This also demonstrates how, in disorders of the skeletal muscle, the striated muscles of the pharynx are affected by sarcomeric weakness despite their intact innervation and central nervous system control, resulting in reduced pharyngeal peristaltic contractions and pathologically prolonged bolus passage. The demonstration of deficits in the oral phase in addition to dysfunction of the UES in IBM patients might explain why exercises mainly aiming at opening of the UES were shown to be ineffective [21]. As training is yet the only method to slow progression of muscle weakness in IBM [22], training of the involved orofacial muscles (e.g., submental muscles or the masseter) could be explored in future studies. Laryngeal elevation in IBM and OPMD was not significantly different to our reference normal values, which is in line with previous reports [2, 8]. Table S3 provides an overview of all RT‐MRI swallowing outcomes from this study and previous studies that used the same MRI technique [2, 19]. It shows relatively consistent results for OTT, PTT, EOT, and LAT in healthy controls and IBM patients across different cohorts.

Previous reports described a frequency of CPB in 39%–41% of IBM patients [7, 23] and 27%–45% of OPMD patients [5, 6], in our study the rate of detection by RT‐MRI was comparatively higher with 80% (12/15) of IBM patients and 54% (7/13) of OPMD patients. Interestingly, the presence of a CPB was related to a prolonged PTT in IBM but not in OPMD, although the CPB size and UES opening diameter were comparable in both diseases. Our data support previous studies showing that cricopharyngeal dysfunction is a key component of obstruction‐related dysphagia in IBM [2, 7, 8, 24, 25, 26, 27, 28], but we found no association between a CPB and pharyngeal bolus passage in OPMD. Biopsies of the cricopharyngeal muscle in IBM patients show fibre necrosis and marked inflammatory cell infiltration [24, 27, 28], whereas in OPMD a predominately dystrophic pattern is described [29]. This might lead to different biomechanical properties of the affected cricopharyngeal muscle tissue, which results in reduced relaxation and a more obstructive effect in IBM. Nonetheless, the successful use of treatment options targeting cricopharyngeal dysfunction like botulinum toxin injection and myotomy has been reported in both IBM and OPMD [24, 25, 27, 28, 30], although not every patient benefited from treatment and some showed reoccurrence of dysphagia after initial treatment response. Therefore, the role of cricopharyngeal dysfunction in dysphagia remains complex and further studies with larger patient cohorts and a combination of RT‐MRI with high resolution impedance manometry might prove useful. As CPBs can appear incidentally even in healthy elderly individuals [31] and in a range of neurological disorders [8], a heterogeneous aetiology of CPBs is to be assumed.

If the three imaging modalities used in our study are compared for their effectiveness to discriminate between the two disease groups, T1 mapping is the most efficient classifier, followed by QMUS and RT‐MRI, but the highest classifying abilities were observed if all three techniques were combined. Clinical assessments and FEES confirmed the presence of severe dysphagia in both, IBM and OPMD patients, but failed to differentiate the underlying pattern. We did not perform videofluoroscopy (VF) in this study since our previous work has already demonstrated equal diagnostic abilities of RT‐MRI and VF [2]. However, achieving a high temporal resolution for MRI of only 40 ms (=25 fps), comparable with standard VF, comes at the expense of spatial resolution, which is therefore lower compared with static T1‐weighted images. Additionally, image analysis can be challenging compared with VF because the MRI signal is obtained from a cross‐sectional slice rather than from overlapping structures in an X‐ray projection. These factors may challenge the measurement of bolus movement especially in patients with severe dysphagia where there are multiple incomplete swallows with fragmentation of the bolus into small amounts, requiring appropriate expertise to analyse the data. Furthermore, the vallecula and piriform sinus are not in the field of view on the mid‐sagittal plane on MRI and, therefore, the amount of residues could not be assessed in this study. This would require an additional axial imaging plane, as demonstrated by Olthoff et al. [19], but at the expense of longer scan times. In clinical practice, FEES continues to be the most reliable method for the evaluation of residues, but it cannot provide information on pharyngeal bolus transit and pathologies of the UES such as a CPB. Despite the aforementioned challenges, RT‐MRI technique clearly reveals the severity and pathophysiology in complex phenotypes of dysphagia, aided by the high contrast of pineapple juice containing paramagnetic manganese as an oral contrast agent and the clear definition of anatomical landmarks for quantification of the observed pathology. Furthermore, the technical limitations are outweighed by the apparent advantages of MRI, which include the absence of radiation and the visualization of soft tissues.

Another relevant difference is the upright body position for FEES and VF versus the supine position for MRI. A study using high‐resolution manometry in healthy subjects demonstrated a significantly higher velopharyngeal pressure generated in the supine position compared with upright, likely representing an adaptive mechanism to prevent bolus material from entering the nasal airway; while only subtle variations of pressure generation were observed in the mesopharynx and UES depending on the body position [32]. These adaptations may influence bolus passage measurement using RT‐MRI, with the supine position potentially revealing earlier signs of dysphagia because muscle weakness impedes a compensatory increase in pharyngeal pressure generation. For the same reason, patients with severe dysphagia were excluded from the present study in order to avoid a potentially increased risk of aspiration in the supine position. Further research is needed to assess the impact of body position on swallowing in patients with dysphagia.

This study presents a comparative analysis between quantitative ultrasound and MRI for characterizing structural abnormalities of orofacial muscles. Previous studies have shown a high correlation between the degree of fatty infiltration on MRI and ultrasound echogenicity in leg muscles of patients with muscular dystrophy [33, 34]. We found that T1 mapping and QMUS correlate positively regarding tongue muscles (Spearman's *r* = 0.61), but not for mouth base and masseter, possibly due to technical limitations of T1 mapping such as the relatively low resolution and difficulties to map small muscles (depending on the observed plane). Also, T1 values can be increased in inflamed muscle and decreased in case of fatty infiltration [35], so the T1 values can be seemingly normal in muscles with both inflammation and fatty infiltration. The QMUS and T1 mapping abnormalities in OPMD are in accordance with previous muscle MRI and ultrasound studies that show fatty infiltration of the tongue as one of the earliest and most prominent signs of the disease [36, 37, 38]. Involvement of the masseter and temporal muscle in OPMD has previously been reported in a CT and MRI study [39].

A direct correlation between some functional and morphometrical findings is lacking in our study. For example, although the tongue is similarly weak in IBM and OPMD, QMUS and T1 mapping do not show abnormalities in the tongue in IBM patients. Possible explanations for the discordance between function and morphometrics could be atrophy of the tongue, fatigue at the moment of clinical testing with underestimation of the force, additional sarcomeric weakness [40], and, specifically for T1 mapping, the above mentioned effect of parallel inflammation and fatty infiltration. This finding should be an important consideration in the selection of outcome measures in upcoming clinical trials.

In conclusion, this study demonstrates the added value of RT‐MRI and QMUS in the characterization of distinct patterns of dysphagia in two different neuromuscular disorders, which could not be achieved by standard diagnostics. Nonetheless, swallowing questionnaires, logopaedic assessments and FEES are useful screening tools for the detection of dysphagia before considering further characterization using high‐resolution imaging by ultrasound or RT‐MRI. RT‐MRI has the advantage of being radiation‐free and noninvasive compared with other functional swallowing assessments such as videofluoroscopy and manometry. QMUS and quantitative T1 mapping both offer potential outcome measures for tracking pathological tissue alterations in clinical trials. These innovative diagnostic tools widen the spectrum for diagnostics, insight into pathophysiological mechanisms, follow‐up examination and further research on the development of treatments for dysphagia in neuromuscular disorders.

## Funding

The study was partly funded by the Association Française contre les Myopathies (AFM‐Téléthon; grant number: 20301) and by the German patient support group Deutsche Gesellschaft für Muskelkranke (DGM; grant number: Sc21/1).

## Ethics Statement

The study was conducted in accordance with the Declaration of Helsinki in its latest version and approval of the local ethics committee was obtained in both participating centers (file numbers 2016‐2928 and 2/7/17). All persons gave their informed consent prior to participation in the study.

## Conflicts of Interest

The authors declare no conflicts of interest.

## Supporting information


**Table S1:** Results of swallowing assessments. Comparison between the two groups was made by independent *t*‐test.
**Table S2:** Results of Flexible endoscopic evaluation of swallowing (FEES). Comparison between the two groups was made by independent *t*‐test.
**Table S3:** Outcomes of real‐time swallowing MRI from this work and previous studies that used the same MRI technique.

**Figure S1:** Heatmap of results from quantitative muscle ultrasound.

**Movie S1:** Movie of real‐time swallowing MRI at a temporal resolution of 25 fps in a 71 year‐old female IBM patient.

**Movie S2:** Movie of real‐time swallowing MRI at a temporal resolution of 25 fps in a 54 year‐old female OPMD patient.

**Movie S3:** Movie of real‐time swallowing MRI at a temporal resolution of 25 fps in a 35 year‐old male healthy control.

**Appendix S1:** (Methods) Swallowing‐related questionnaire and swallowing assessments.
**Appendix S2:** (Methods) Flexible endoscopic evaluation of swallowing (FEES).
**Appendix S3:** (Methods) Clinical evaluation of muscle force (MRC).
**Appendix S4:** Anti‐cN‐1A autoantibody status in IBM patients.
Supporting Information References.
