# Supplementary material for: Evaluation of Dysphagia in Myositis and Muscular Dystrophy Using Real‐Time MRI and Quantitative Muscle Ultrasound
Source: J Cachexia Sarcopenia Muscle. 2026 Mar 13;17(2):e70187. doi: 10.1002/jcsm.70187 (PMC13140979; doi:10.1002/jcsm.70187)
Supplement: Supplementary file 1 — Table S1: Results of swallowing assessments. Comparison between the two groups was made by independent t‐test. Table S2: Results of Flexible endoscopic evaluation of swallowing (FEES). Comparison between the two groups was made by independent t‐test. Table S3: Outcomes of real‐time swallowing MRI from this work and previous studies that used the same MRI technique. [file JCSM-17-e70187-s003.pdf]

Manuscript title: Evaluation of dysphagia in myositis and muscular dystrophy using real-time MRI and quantitative muscle ultrasound

Authors

Rachel Zeng, MD*, Anke Rietveld, MD*, Omar Al-Bourini, MD*, Rosemarie H.M.J.M. Kroon, MA, Arno Olthoff, MD, Matthias Weidenmüller, MD, Per-Ole Carstens, MD, Isabel Kommerell, Saskia G. Schütz, MD, Corinne G.C. Horlings, MD, Johanna G. Kalf, PhD, Bert J.M. de Swart, PhD, Baziel G.M. van Engelen, MD, Tim Friede, PhD, Sabine Hofer, PhD, Jens Frahm, PhD, Ali Seif Amir Hosseini, MD**, Jens Schmidt, MD**, Christiaan G.J. Saris, MD**

* shared first authorship **shared last authorship

Correspondence to:

Christiaan GJ Saris, MD
Department of Neurology
Radboudumc Research Institute for Medical Innovation
Geert Grooteplein 10
6500 HB, Nijmegen, The Netherlands
E.mail: c.saris@radboudumc.nl
Tel: +31 (0)24-3616600

Correspondence may also be addressed to:
Jens Schmidt, MD, FEAN, FAAN
Department of Neurology and Pain Treatment
Neuromuscular Center - Center for Translational Medicine
Immanuel Klinik Rüdersdorf, University Hospital of the Brandenburg Medical School
Seebad 82/83
15562 Rüdersdorf bei Berlin, Germany
E-Mail: j.schmidt@gmx.org

Supporting Information Tables

Table S1: Results of swallowing assessments. Comparison between the two groups was made by independent t-test.

	**IBM** Mean (SD)	**OPMD** Mean (SD)	**IBM vs. OPMD** Mean difference (95% CI)	**IBM vs. OPMD *p***	**Healthy controls** Mean (SD)	**IBM and OPMD vs. controls** Mean difference (95% CI)	**IBM and OPMD vs. controls *p***
**Swallowing – clinical assessment** §*Data missing from 1 IBM patient who could not perform a sustained movement of the tongue*							
Maximum swallowing volume test (mL)	27.8 (23.1)	37.3 (17.3)	-9.5 (-25.1 – 6.0)	0.22	53.8 (20.3)	22.0 (13.8 – 30.2)	**<0.001**
Maximum swallowing speed (mL/s)	9.6 (6.5)	14.1 (7.3)	-4.5 (-9.6 – 0.6)	0.08	25.0 (9.9)	13.6 (10.5 – 16.6)	**<0.001**
Maximum isometric tongue pressure anterior (kPa)	43.9 (15.6)	35.6 (10.3)	8.3 (-1.9 – 18.5)	0.11	50.4 (13.6)	9.9 (4.5 – 15.3)	**<0.001**
Maximum isometric tongue pressure posterior (kPa)	37.3 (16.3)	34.5 (12.2)	2.8 (-8.2 – 13.8)	0.60	49.7 (14.2)	13.6 (8.0 – 19.3)	**<0.001**
Maximum endurance anterior (s)§	18.7 (16.2)	16.0 (8.9)	2.7 (-7.5 – 12.9)	0.59	18.6 (13.1)	1.0 (-4.2 – 6.3)	0.70
Maximum endurance posterior (s)§	14.3 (12.9)	12.0 (8.8)	2.3 (-6.3 – 11.0)	0.59	12.8 (11.7)	-3.4 (-11.0 – 4.1)	0.36
**Chewing – clinical assessment** **Data missing from 3 IBM patients and 1 OPMD patient who were unable to chew and swallow a cracker*							
Maximum bite force (kg)	15.8 (10.7)	17.4 (6.3)	-1.6 (-8.4 – 5.2)	0.63	16.2 (8.7)	-0.3 (-3.8 – 3.2)	0.86
Test of Masticating and Swallowing Solids chewing time (s)*	67.8 (47.6)	45.6 (10.8)	22.3 (-6.7 – 51.3)	0.13	34.1 (15.5)	-23.8 (-38.9 – -8.7)	**0.003**
**Speaking – clinical assessment**							

Maximum phonation time (s)	16.6 (9.5)	15.3 (7.3)	1.3 (-5.1 – 7.8)	0.68	22.0 (9.9)	6.0 (2.1 – 9.8)	**0.003**

Table S2: Results of Flexible endoscopic evaluation of swallowing (FEES). Comparison between the two groups was made by independent t-test.

	**IBM**	**OPMD**	***p***
Secretion (Murray)	0 = 5 (33.3%)<br>1 = 5 (33.3%)<br>2 = 5 (33.3%)	0 = 2 (15,4%)<br>1 = 9 (69.2%)<br>2 = 2 (15.4%)	0.40<br>(normal vs. pharyngeal/inconsistent)
Bolus-Leaking	no = 15 (100%)<br>yes = 0 (0%)	no = 12 (92.3%)<br>yes = 1 (7.7%)	0.46
Bolus-Retention	no = 3 (20%)<br>yes = 12 (80%)	no = 0 (0%)<br>yes = 13 (100%)	0.23
Penetration/Aspiration (Rosenbek scale), (mean (SD))	4.0 (1.5)	4.0 (2.1)	0.60
Data missing from 1 of the 16 IBM patients (did not tolerate the assessment)<br>*Secretion (Murray): 0 normal, 1 pharyngeal, 2 inconsistent (larynx/pharynx)*<br>*Penetration/Aspiration (Rosenbek): range 3 – 8 (3 = normal, 8 = silent aspiration)*			

Table S3: Outcomes of real-time swallowing MRI from this work and previous studies that used the same MRI technique. OTT, oral transit time; PTT, pharyngeal transit time; PCT, pharyngeal constriction time; EOT, esophageal opening time; HMA, hyoid-mental approximation; LAT, laryngeal elevation; CPB, cricopharyngeal bar.

	**IBM**<br>n=16[1]<br>n=20[2]	**OPMD**<br>n=13[1]	**Healthy controls**<br>n=22[1]<br>n=10[3]
OTT (ms)	440 ± 221[1]<br>411 ± 200[2]	854 ± 801[1]	273 ± 74[1]<br>200 ± 83[3]
PTT (ms)	1785 ± 1468[1]<br>1579 ± 1561[2]	1051 ± 628[1]	653 ± 125[1]<br>467 ± 117[3]
PCT (ms)	2243 ± 1471[1]	1394 ± 1075[1]	1102 ± 808[1]<br>410 ± 73[3]
EOT (ms)	353 ± 101[1#]<br>324 ± 65[2]	355 ± 109[1]	386 ± 68[1]<br>261 ± 62[3]
LAT abs. (mm)	23.6 ± 6.2[1]<br>30.8 ± 9.01[2]	22.9 ± 5.0[1]	26.5 ± 4.7[1]
LAT in relation to C2-C4 distance (%)	64 ± 15.0[1]	64 ± 13.3[1]	72.1 ± 11.0[1]
HMA (%)	19.9 ± 9.9[1]	20.9 ± 6.0[1]	29.8 ± 7.9[1]
Esophageal sphincter opening diameter (mm)**	4.2 ± 0.7[1]	4.4 ± 0.8[1]	4.5 ± 0.9[1]
CP occurrence	80% (12/15) [1#]<br>83% (15/18)[2##]	54% (7/13) [1]	0 (0/22) [1]
CP diameter (mm)	4.7 ± 1.8[1]<br>5.5 ± 3.4[2]	4.9 ± 1.5[1]	n.a.

[1]This work

[2]Olthoff A, Carstens P, Zhang S, et al. Evaluation of dysphagia by novel real-time MRI. Neurology. 2016:2132–8.

[3]Olthoff A, Zhang S, Schweizer R, Frahm J. On the physiology of normal swallowing as revealed by magnetic resonance imaging in real time. Gastroenterol Res Pract. 2014;2014:493174.

[#]CPB could not be analyzed in one IBM patient due to imaging artefacts

[##] CPB could not be analyzed in two IBM patients due to technical limitations
