# Supplementary material for: Evaluation of Dysphagia in Myositis and Muscular Dystrophy Using Real‐Time MRI and Quantitative Muscle Ultrasound
Source: J Cachexia Sarcopenia Muscle. 2026 Mar 13;17(2):e70187. doi: 10.1002/jcsm.70187 (PMC13140979; doi:10.1002/jcsm.70187)
Supplement: Supplementary file 2 — Figure S1: Heatmap of results from quantitative muscle ultrasound. [file JCSM-17-e70187-s001.pdf]

Manuscript title: Evaluation of dysphagia in myositis and muscular dystrophy using real-time MRI and quantitative muscle ultrasound

Authors
Rachel Zeng, MD*, Anke Rietveld, MD*, Omar Al-Bourini, MD*, Rosemarie H.M.J.M. Kroon, MA, Arno Olthoff, MD, Matthias Weidenmüller, MD, Per-Ole Carstens, MD, Isabel Kommerell, Saskia G. Schütz, MD, Corinne G.C. Horlings, MD, Johanna G. Kalf, PhD, Bert J.M. de Swart, PhD, Baziel G.M. van Engelen, MD, Tim Friede, PhD, Sabine Hofer, PhD, Jens Frahm, PhD, Ali Seif Amir Hosseini, MD**, Jens Schmidt, MD**, Christiaan G.J. Saris, MD**
* shared first authorship **shared last authorship

Correspondence to:
Christiaan GJ Saris, MD
Department of Neurology
Radboudumc Research Institute for Medical Innovation
Geert Grooteplein 10
6500 HB, Nijmegen, The Netherlands
E.mail: c.saris@radboudumc.nl
Tel: +31 (0)24-3616600

Correspondence may also be addressed to:
Jens Schmidt, MD, FEAN, FAAN
Department of Neurology and Pain Treatment
Neuromuscular Center - Center for Translational Medicine
Immanuel Klinik Rüdersdorf, University Hospital of the Brandenburg Medical School
Seebad 82/83
15562 Rüdersdorf bei Berlin, Germany
E-Mail: j.schmidt@gmx.org

Supporting Information Figures

Figure S1: Heatmap of results from quantitative muscle ultrasound

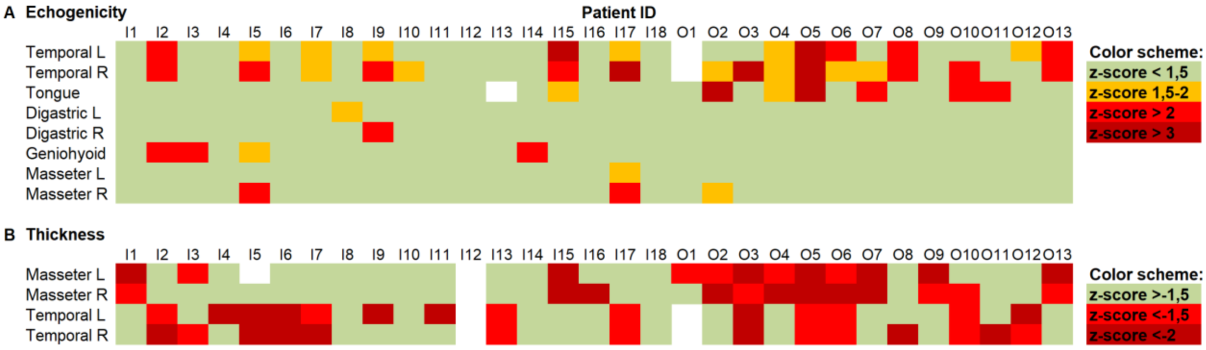

Heatmap of echogenicity (A) and muscle thickness (B) obtained by QMU. I1-I18 are individual IBM patients, O1-O13 are individuals with OPMD. Color codes represent z-scores, with echogenicity z-scores between 1.5 and 2 considered borderline abnormal, z-scores ≥ 2 considered abnormal and z-scores > 3 considered as severely abnormal. Muscle thickness z-scores ≤ -1.5 were considered abnormal. White represents missing data.
