# Supplementary material for: Evaluation of Dysphagia in Myositis and Muscular Dystrophy Using Real‐Time MRI and Quantitative Muscle Ultrasound
Source: J Cachexia Sarcopenia Muscle. 2026 Mar 13;17(2):e70187. doi: 10.1002/jcsm.70187 (PMC13140979; doi:10.1002/jcsm.70187)
Supplement: Supplementary file 6 — Appendix S1: (Methods) Swallowing‐related questionnaire and swallowing assessments. Appendix S2: (Methods) Flexible endoscopic evaluation of swallowing (FEES). Appendix S3: (Methods) Clinical evaluation of muscle force (MRC). Appendix S4: Anti‐cN‐1A autoantibody status in IBM patients. Supporting Information References. [file JCSM-17-e70187-s004.pdf]

**Manuscript title: Evaluation of dysphagia in myositis and muscular dystrophy using real-time MRI and quantitative muscle ultrasound**

**Authors**
Rachel Zeng, MD*, Anke Rietveld, MD*, Omar Al-Bourini, MD*, Rosemarie H.M.J.M. Kroon, MA, Arno Olthoff, MD, Matthias Weidenmüller, MD, Per-Ole Carstens, MD, Isabel Kommerell, Saskia G. Schütz, MD, Corinne G.C. Horlings, MD, Johanna G. Kalf, PhD, Bert J.M. de Swart, PhD, Baziel G.M. van Engelen, MD, Tim Friede, PhD, Sabine Hofer, PhD, Jens Frahm, PhD, Ali Seif Amir Hosseini, MD**, Jens Schmidt, MD**, Christiaan G.J. Saris, MD**
* shared first authorship **shared last authorship

**Correspondence to:**
Christiaan GJ Saris, MD
Department of Neurology
Radboudumc Research Institute for Medical Innovation
Geert Grooteplein 10
6500 HB, Nijmegen, The Netherlands
E.mail: c.saris@radboudumc.nl
Tel: +31 (0)24-3616600

Correspondence may also be addressed to:
Jens Schmidt, MD, FEAN, FAAN
Department of Neurology and Pain Treatment
Neuromuscular Center - Center for Translational Medicine
Immanuel Klinik Rüdersdorf, University Hospital of the Brandenburg Medical School
Seebad 82/83
15562 Rüdersdorf bei Berlin, Germany
E-Mail: j.schmidt@gmx.org

## Supporting Information Appendix

### Appendix S1: Swallowing-related questionnaire and swallowing assessments

The Swallowing Related Quality of Life (SWAL-QoL) questionnaire was completed by each patient [S1, S2]. Swallowing assessments were performed by an experienced speech therapist (RK) according to standard procedures. The results of the swallowing assessment were compared with norm values of age-matched healthy controls from our local database [S3]. All patients underwent the following series of swallowing assessments:

- Maximum swallowing speed: the time in seconds a patient needs to swallow 150 ml of water as quickly as possible [S4]. Patients who were unable to perform this test were considered having a swallowing speed of 0 ml/second.
- Maximum volume test: the maximum volume of water in milliliters that a patient can take in one swallow [S5].

- Maximum bite force (measured in Newton and converted to kilograms to allow comparison with previous publications), measured with the Bite Force Gauge of the VU University, Amsterdam, The Netherlands [S6].
- Maximum tongue strength (in kilopascal, kPa) and tongue endurance (in seconds): measured using the Iowa Oral Performance Instrument (IOPI Medical, Redmond, WA [S7]. First, patients were instructed to push the bulb on the tongue against the roof of the mouth as hard as possible (anterior and posterior strength). Secondly, patients were asked to push the bulb on the tongue against the roof of the mouth as long as possible. Three trials of bite force, tongue strength and endurance were measured in each patient, the best performance was retained for analysis.
- Maximum chewing time: chewing and swallowing a standardized cracker as quickly as possible, evaluated by the Test of Masticating and Swallowing Solids chewing time (TOMASS) protocol [S8].
- Maximum phonation time: the number of seconds a patient can produce a sustained ‘ah’ [S9].

**Appendix S2: Flexible endoscopic evaluation of swallowing (FEES)**

Transnasal FEES was performed using a flexible endoscope (Olympus ENF, Hamburg, Germany) which was connected to a camera (Olympus visera OTV-S7). Patients were examined in an upright seated position and the assessments were videorecorded with a typical temporal resolution of 25 fps. An oral bolus of 1 teaspoon green pear compote was used to ensure a clear contrast to the tissues of the oropharyngeal tract. All videos were stored on a hard disk (rpSzene; Rehder & Partner GmbH, Hamburg, Germany) for subsequent evaluation.

FEES findings were described using the Murray secretion scale (score 0-2) [S10] and Rosenbek’s penetration-aspiration scale (score 1 to 8) [S11].

**Appendix S3: Clinical evaluation of muscle force (MRC)**

Muscle force was assessed in all study participants using the Medical Research Council (MRC) scale [S12]. The MRC sum score with a maximum of 130, sums up MRC values of shoulder abduction, neck extension and flexion, elbow extension and flexion, wrist extension and flexion, finger flexion, hip flexion and adduction, knee extension and flexion, foot extension and flexion. In our study cohort, IBM patients had a lower MRC sum score compared to OPMD patients (103.3 ± 17.1 versus 126.9 ± 4.4, $p < 0.001$).

**Appendix S4: Anti-cN-1A autoantibody status in IBM patients**

IBM patients underwent anti-cN-1A autoantibody detection using full-length cN-1A ELISA (Euroimmun AG, order number EA 1675-4801G). Anti-cN-1A autoantibodies were detected in 12/18 IBM patients (67%). The baseline characteristics (age, sex and duration of symptoms), SWAL-QoL score, MRC sum score, FEES, QMUS and real-time MRI results did not differ between anti-cN-1A positive and -negative patients (data not shown). The other swallowing assessments showed comparable results for anti-cN-1A seropositive and -negative patients.

**Supporting Information References**


S1. Bogaardt HCA, Speyer R, Baijens LWJ, Fokkens WJ. Cross-cultural adaptation and validation of the Dutch version of SWAL-QoL. Dysphagia. 2009;24:66–70. doi:10.1007/s00455-008-9174-z.

S2. McHorney CA, Robbins J, Lomax K, Rosenbek JC, Chignell K, Kramer AE, Bricker DE. The SWAL-QOL and SWAL-CARE outcomes tool for oropharyngeal dysphagia in adults: III. Documentation of reliability and validity. Dysphagia. 2002;17:97–114. doi:10.1007/s00455-001-0109-1.

S3. Kalf H. Twee kwantitatieve sliktests: Eenvoudig objectief slikonderzoek. Logopedie en foniatrie. 2004:640–6.

S4. Nathadwarawala KM, Nicklin J, Wiles CM. A timed test of swallowing capacity for neurological patients. J Neurol Neurosurg Psychiatry. 1992;55:822–5. doi:10.1136/jnnp.55.9.822.

S5. Kalf H, Weikamp JG, Swart BJ de. Normal values of the swallowing volume test (SVT). 8th CPLOL congress, The Hague, the Netherlands, 2012 [abstract].

S6. Weijenberg RAF, Lobbezoo F, Knol DL, Tomassen J, Scherder EJA. Increased masticatory activity and quality of life in elderly persons with dementia--a longitudinal matched cluster randomized single-blind multicenter intervention study. BMC Neurol. 2013;13:26. doi:10.1186/1471-2377-13-26.

S7. Clark HM, Solomon NP. Age and sex differences in orofacial strength. Dysphagia. 2012;27:2–9. doi:10.1007/s00455-011-9328-2.

S8. Huckabee M-L, McIntosh T, Fuller L, Curry M, Thomas P, Walshe M, et al. The Test of Masticating and Swallowing Solids (TOMASS): reliability, validity and international normative data. Int J Lang Commun Disord. 2018;53:144–56. doi:10.1111/1460-6984.12332.

S9. Speyer R, Bogaardt HCA, Passos VL, Roodenburg NPHD, Zumach A, Heijnen MAM, et al. Maximum phonation time: variability and reliability. J Voice. 2010;24:281–4. doi:10.1016/j.jvoice.2008.10.004.

S10. Murray J, Langmore SE, Ginsberg S, Dostie A. The significance of accumulated oropharyngeal secretions and swallowing frequency in predicting aspiration. Dysphagia. 1996;11:99–103. doi:10.1007/BF00417898.

S11. Rosenbek JC, Robbins JA, Roecker EB, Coyle JL, Wood JL. A penetration-aspiration scale. Dysphagia. 1996;11:93–8. doi:10.1007/BF00417897.

S12. Compston A. Aids to the investigation of peripheral nerve injuries. Medical Research Council: Nerve Injuries Research Committee. His Majesty's Stationery Office: 1942; pp. 48 (iii) and 74 figures and 7 diagrams; with aids to the examination of the peripheral nervous system. By Michael O'Brien for the Guarantors of Brain. Saunders Elsevier: 2010; pp. 8 64 and 94 Figures. Brain. 2010;133:2838–44. doi:10.1093/brain/awq270.
